# Position Optimization by Stand Adjustment in Transjugular Transcatheter Tricuspid Valve Replacement: The POSA-tTVR Technique

**DOI:** 10.1016/j.jscai.2026.104258

**Published:** 2026-02-10

**Authors:** Vyanne H.T. Chan, Kevin K.H. Kam, Fuk Kei Fong, Geri L.N. Wong, Alex P.W. Lee, Kent C.Y. So

**Affiliations:** a Division of Cardiology, Department of Medicine and Therapeutics, Prince of Wales Hospital, Chinese University of Hong Kong, Hong Kong SAR, China; b Li Ka Shing Institute of Health Sciences, The Chinese University of Hong Kong, Hong Kong SAR, China

**Keywords:** innovation, technique, transcatheter tricuspid valve replacement, tricuspid regurgitation

**Figure 1:**
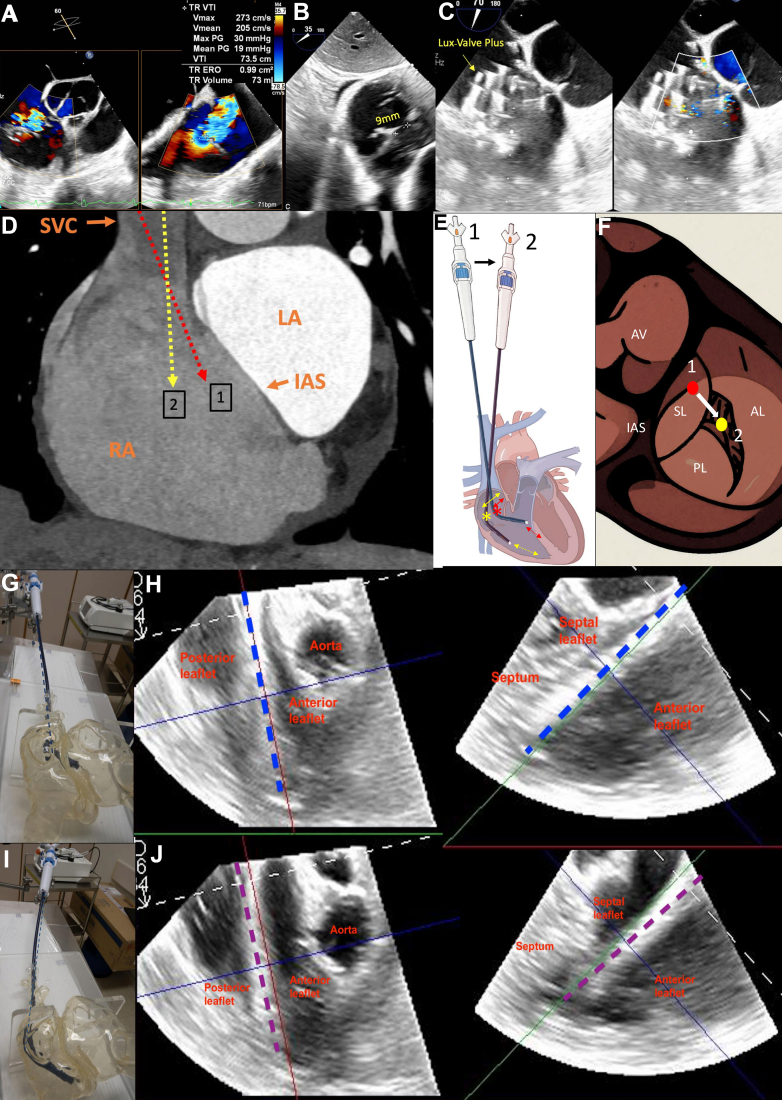
(**A**) Preprocedural transesophageal echocardiography with torrential tricuspid regurgitation with posteroseptal and anteroseptal jets. (**B**). Wide coaptation gap of 9 mm. (**C**). Postprocedure transesophageal echocardiography showing LuX-Valve Plus deployed with coaxial alignment (yellow arrow), and color Doppler showing the elimination of tricuspid regurgitation. (**D**). Reconstructed–computed tomography image showed superior vena cava entry (orange arrow) directing delivery system to anteromedial right atrium and cause septal hugging at position (1, red dotted line) and medial stand positioning reduces septal hugging at position (2, yellow dotted line). (**E**). Schematic illustration of medial and lateral stand positioning and corresponding pivot point changes during POSA-tTVR manipulation. Delivery stand movement from lateral (1) to medial (2). Asterisks denote pivot points. In position (1), the system shows septal hugging (red double-sided arrow) and deeper right ventricular entry (red–double-sided dotted arrow). In position (2), the system is redirected away from the septum (yellow double-sided arrow) and reduces RV depth (yellow–double-sided dotted arrow). (**F**). Illustration showing pivot point positions relative to the tricuspid valve at position (1, red circle) and (2, yellow circle). (**G**). Bench model showing initial stand position before POSA-tTVR manipulation. (**H**). Transesophageal echocardiography showing delivery tip malaligned due to septal hugging before POSA-tTVR (dotted blue line). (**I**). Bench model showing stand position moving medial after POSA-tTVR. (**J**). Transesophageal echocardiography showing coaxial alignment achieved with the medial stand repositioning after POSA-tTVR (purple dotted line), and septal hugging was eliminated. AL, anterior leaflet; AV, aortic valve; IAS, interatrial septum; LA, left atrium; PL, posterior leaflet; RA, right atrium; SL, septal leaflet; SVC, superior vena cava.

A 59-year-old woman with surgically inoperable tricuspid regurgitation presented with right heart failure. Transesophageal echocardiography showed a wide coaptation gap (9 mm) with dominant posteroseptal and additional anteroseptal jets, making her unsuitable for transcatheter edge-to-edge repair (Figure 1A, B). After a multidisciplinary heart team discussion, transjugular transcatheter tricuspid valve replacement (TTVR) using LuX-Valve Plus (Jenscare Scientific) was planned.

During implantation via the right internal jugular vein, device alignment was challenging because of the superior vena cava entry directing the delivery system toward the anteromedial right atrium, resulting in “septal hugging” (Figure 1D). The existing delivery system lacks a “septal/lateral” steering knob, limiting medial-lateral adjustment. To overcome this, the Position Optimization by Stand Adjustment in transjugular Transcatheter Tricuspid Valve Replacement (POSA-tTVR) technique was applied, which involves over-adjustment of the stabilizer platform toward an extreme medial position (Supplemental Video 1). This maneuver shifted the pivot point laterally and reduced “septal hugging,” hence allowing the delivery system to align perpendicularly to the tricuspid annulus (Figure 1D). The LuX-Valve Plus was then successfully deployed at optimal alignment, resulting in complete tricuspid regurgitation elimination (Figure 1C).

The POSA-tTVR technique allows both medial and lateral stand adjustments to address different anatomic challenges during transjugular valve implantation:

1. Medial stand positioning shifts the pivot point laterally, reduces septal hugging, and helps gain height above the tricuspid annulus. It is particularly beneficial in cases where superior vena cava entry directs the system toward the septum or in patients with a small right ventricle (Figure 1E, F, I, J; Supplemental Videos 1 and 2).
2. Lateral stand positioning shifts the pivot medially and helps shed height from the tricuspid valve annular plane and steer the system deeper into the right ventricle. This is beneficial in patients with a large right atrium (Figure 1E-H; Supplemental Videos 3 and 4).

For illustration, a bench model and a schematic diagram are included to demonstrate the POSA-tTVR maneuver and its effect on system orientation and valve alignment (Supplemental Videos 3 and 4; Figure E, F).

This approach leverages the position of vascular entry into the right atrium to directly adjust the pivot point, thereby improving coaxiality and device positioning in anatomically challenging cases. Compared with the transfemoral TTVR, the transjugular approach offers greater flexibility for stand maneuvers, and this represents a distinct advantage over the transfemoral route in selected cases. In addition to the Lux-Valve Plus platform, transjugular TTVR is also gaining wider adoption with other devices such as Evoque (Edwards Lifesciences).^1^ The POSA-tTVR maneuver can serve as a broadly applicable and reproducible technique to enhance alignment and procedural success across multiple valve platforms.
